# CD137 Facilitates the Resolution of Acute DSS-Induced Colonic Inflammation in Mice

**DOI:** 10.1371/journal.pone.0073277

**Published:** 2013-09-04

**Authors:** Julia M. Martínez Gómez, Lieping Chen, Herbert Schwarz, Thomas Karrasch

**Affiliations:** 1 Department of Microbiology, Yong Loo Lin School of Medicine, National University of Singapore, Singapore, Singapore; 2 Department of Physiology, Yong Loo Lin School of Medicine, National University of Singapore, Singapore, Singapore; 3 Department of Immunobiology, Yale University School of Medicine, New Haven, Connecticut, United States of America; 4 Department of Internal Medicine I, University of Regensburg, Regensburg, Germany; University of Chicago, United States of America

## Abstract

**Background:**

CD137 and its ligand (CD137L) are potent immunoregulatory molecules that influence activation, proliferation, differentiation and cell death of leukocytes. Expression of CD137 is upregulated in the lamina propria cells of Crohn’s disease patients. Here, the role of CD137 in acute Dextran-Sodium-Sulfate (DSS)-induced colitis in mice was examined.

**Methods:**

We induced acute large bowel inflammation (colitis) via DSS administration in CD137^−/−^ and wild-type (WT) mice. Colitis severity was evaluated by clinical parameters (weight loss), cytokine secretion in colon segment cultures, and scoring of histological inflammatory parameters. Additionally, populations of lamina propria mononuclear cells (LPMNC) and intraepithelial lymphocytes (IEL) were characterized by flow cytometry. In a subset of mice, resolution of intestinal inflammation was evaluated 3 and 7 days after withdrawal of DSS.

**Results:**

We found that both CD137^−/−^ and WT mice demonstrated a similar degree of inflammation after 5 days of DSS exposure. However, the resolution of colonic inflammation was impaired in the absence of CD137. This was accompanied by a higher histological score of inflammation, and increased release of the pro-inflammatory mediators granulocyte macrophage colony-stimulating factor (GM-CSF), CXCL1, IL-17 and IFN-γ. Further, there were significantly more neutrophils among the LPMNC of CD137^−/−^ mice, and reduced numbers of macrophages among the IEL.

**Conclusion:**

We conclude that CD137 plays an essential role in the resolution of acute DSS-induced intestinal inflammation in mice.

## Introduction

CD137 (TNFRS9, 4-1BB) is a member of the tumor necrosis factor (TNF) receptor family, and CD137 ligand (CD137L) is a member of the TNF superfamily. CD137 and its ligand are expressed mostly on immune cells, and the CD137 receptor/ligand system regulates activation, proliferation, differentiation and cell death of leukocytes [1], [2], [3], [4]. CD137 is expressed activation-dependently on T cells, where it acts as a potent costimulatory molecule [5]. Crosslinking of CD137 enhances T cell activity sufficiently to induce rejection of tumors or transplants [6], [7]. Surprisingly, the very same agonistic anti-CD137 antibodies have been demonstrated to ameliorate disease severity in various murine models of autoimmune diseases [8], [9], [10].

Human inflammatory bowel diseases (IBD) are autoimmune diseases that are characterized by chronic autoinflammatory processes within the intestinal mucosal lamina propria. TNF has been demonstrated to play a key role in human IBD [11], [12], [13] and in experimental models of autoimmune colitis in mice [14], [15], where TNF induces intestinal epithelial cell apoptosis during intestinal inflammation, thereby aggravating the disease [16]. Of note, CD137L signaling has been demonstrated to induce TNF secretion [17], [18], and TNF receptor 1 (TNFR1) acts as a coreceptor for CD137L and mediates CD137L signaling [19]. CD137 is induced in human IBD and in murine models of intestinal inflammation [20], [21]. However, the impact of CD137 and CD137L on experimental colitis in mice as well as in human IBD is not known.

In the current study, we induced acute large bowel inflammation (colitis) via Dextran Sodium Sulfate (DSS) exposure in CD137^−/−^ and WT mice. We found that while both CD137^−/−^ and WT mice demonstrated a similar degree of inflammation after 5 days of DSS exposure, the resolution of colonic inflammation was significantly impaired in CD137^−/−^ mice. This was accompanied by increased histological signs of inflammation, increased levels of the pro-inflammatory mediators GM-CSF, CXCL1, IL-17 and IFN-γ as well as increased neutrophil recruitment and reduced macrophage numbers in the colonic lamina propria of CD137^−/−^ mice.

## Materials and Methods

### Mice

C57BL/6 mice were obtained from the Centre for Animal Resources of the National University of Singapore. Generation of CD137^−/−^ mice on a C57Bl/6 background has been described previously [22]. CD137^−/−^ mice were bred in-house under specific pathogen-free (SPF) conditions. All experiments were conducted at the National University of Singapore. The experimental protocol was approved by the National University of Singapore Institutional Animal Care and Use Committee (NUS IACUC). Institutional guidelines for animal care and use were followed throughout the experiments. At the designated time points for cell or tissue harvesting, mice were sacrificed by CO_2_-asphyxiation. During the course of DSS treatment, mice were checked daily for signs of distress or advanced colitis: Animals losing above 20% of their original body weight or showing signs of distress (e.g. apathy, aggressive behavior upon contact, unphysiological bodily posture, shaggy fur) were sacrificed immediately by CO_2_-asphyxiation. No additional analgetics were used during the course of the experiments.

### DSS-induced Acute Colitis in Mice

DSS-induced acute colitis in mice is a well-established model of human IBD [23], [24]. In pilot experiments, six to eight week-old CD137^−/−^ and wild-type (WT) mice were administered 3.5% Dextran Sodium Sulfate (DSS, MW 36.000–50.000, MP Biomedicals) in drinking water ad libitum versus tap water control over a period of 7 days. Water consumption was monitored and was comparable between the different groups. Weight loss, stool consistency as well as stool occult blood tests (Hemocare-Test, Care Diagnostica) were monitored daily to provide an assessment of colitis severity during the experiment. At day 7, mice were sacrificed, the intestines were removed, washed in ice-cold phosphate-buffered saline (PBS), and samples were harvested, fixed in 10% formalin for 24 h and embedded in paraffin to provide sections for histological scoring of inflammation severity.

To study the resolution of inflammation, CD137^−/−^ and WT mice were administered 2.0% DSS in drinking water ad libitum versus tap water control over a period of 5 days. After 5 days of DSS exposure, all mice were switched back to tap water for an additional period of 0 to 7 days. Water consumption was monitored and was comparable between the different groups. Weight loss, stool consistency as well as stool occult blood tests were monitored daily to provide an assessment of colitis severity during the experiment. At the designated time points (day 5, day 8 and day 11 of the experiment), mice were sacrificed, the intestines were removed, washed in ice-cold PBS, and samples were harvested, fixed in 10% formalin for 24 h and embedded in paraffin to provide sections for histological scoring of inflammation severity. Additional samples were harvested for evaluation of cytokine secretion in colonic segment cultures, as described below. Additional sets of mice were sacrificed at the designated time points (day 5 and day 8 of the experiment) for evaluation of lamina propria mononuclear cell compartments, as described below.

### Histological Evaluation of DSS-induced Colitis Severity

Histological evaluation of DSS-induced colitis severity was performed as previously described [25]. Briefly, paraffin-embedded colonic cross-sections were stained with Hematoxylin-Eosin and evaluated under a light microscope by an experienced investigator blinded to the experimental conditions. The distal colon was evaluated since it has been demonstrated to be the most severely affected colon segment in DSS-induced colitis [23], [26], and to provide a score representing the disease severity in the entire colon [27]. Coded sections were scored using a validated scoring system developed by Cooper [23] and Dieleman [28], and modified by Williams [27] using a scale of 0 to 40. For each animal, 2 sections approximately 400 µm apart were scored and averaged.

### Evaluation of Cytokine Secretion in Colon Segment Tissue Cultures

Cytokine secretion was evaluated in cultures of colon segments as previously described by other groups [29], [30]. Briefly, mice were sacrificed, the intestines were removed, opened lengthwise and washed in PBS. After being cut into pieces of approximately 1 cm, colon segments were shaken vigorously for 30 min in PBS. Tissue segments of approximately 50 to 100 mg were then divided into wells of a 24-well tissue culture plate and cultured in triplicates at 37°C for 24 h in 1 ml of complete medium containing penicillin/streptomycin, gentamycin and amphotericin B. Culture supernatants were then collected and stored at −20°C until further evaluation.

Cytokine concentrations in culture supernatants were evaluated using ELISA kits (R&D Systems) according to the manufacturer’s specifications. Cytokine concentrations are expressed per g of colonic tissue.

### Isolation of IEL and LPMNC and Flow Cytometry

Isolation of intestinal lymphocytes from both epithelial (IEL) and lamina propria (LPMNC) compartments of the large intestine was performed as previously described [31], [32]. Briefly, mice were sacrificed, the large intestines were removed, opened lengthwise and washed in PBS. After cutting the colon into small pieces, the intestinal epithelial layer was selectively detached by treatment with 1 mM DTT in a shaker water-bath at 37°C for 30 min, followed by vortexing for 20 sec. The supernatant containing IEL and epithelial cells was then collected.

The residual tissues were incubated in a shaker water-bath at 37°C for 1 h with collagenase D (1 mg/ml) (Roche) and then disrupted mechanically through a 70 µm cell strainer. IEL and LPMNC fractions were further purified by Percoll (Sigma-Aldrich) density gradient centrifugation. Mononuclear cells enriched in the interface between 70 and 30% Percoll were recovered.

Freshly isolated intestinal IEL and LPMNC single cell suspensions were washed in staining buffer (5% FBS/0.02% NaN3 in PBS), blocked with anti-CD16/32 antibody for 5 min at RT and then stained for 30 min at 4°C with the following surface antibodies: anti-CD45-V500 (BD Pharmingen), anti-CD3 A700, anti-CD4 PB (BD Pharmingen), anti-CD8 FITC, anti-CD11b PE, anti-Ly6C APC, anti-Ly6G PE-Cy7, and with a live/dead fixable dead stain kit in the far red fluorescent APC-Cy7 (Invitrogen). Cell suspensions were then washed and analyzed using an LSRFortessa flow cytometer (BD Biosciences) and FlowJo software (Version 9.3.1, Treestar). Unless otherwise stated antibodies were from eBioscience.

### Statistical Analysis

Data are expressed as mean ± SEM. Prism software (Version 5, GraphPad software) was employed to analyze the data using the Student T-test. p<0.05 was considered statistically significant.

## Results

### CD137^−/−^ and WT Mice Develop a Similar Disease Severity in Acute 3.5% DSS-induced Colitis

We first examined the impact of an acute 3.5% DSS challenge in CD137^−/−^ versus WT mice, which induces acute large bowel inflammation [23], [24]. Mice were given 3.5% DSS in drinking water ad libitum for a period of 7 days, after which time mice were sacrificed. The intestines were removed and sections were embedded for histological scoring of inflammation severity. Weight loss, stool consistency as well as stool occult blood tests were used to monitor colitis severity in mice throughout the experiment.

**Figure 1:**
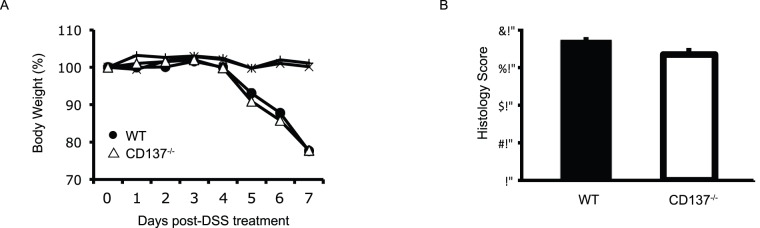
CD137^−/−^ and WT mice develop a similar disease severity in acute 3.5% DSS-induced colitis. A, Percentage of body weight loss over the course of 7 days of treatment with 3.5% DSS. B, Histology score of WT (black bars) and CD137^−/−^ (white bars) mice treated with 3.5% DSS for 7 days. Data are representative of two independent experiments, shown here are means ± SEM, n = 15 for DSS groups, n = 5 for water control groups.

No difference was observed in CD137^−/−^ versus WT mice in colitis severity during the 3.5% DSS challenge as measured by weight loss (Fig. 1A), stool consistency as well as stool occult blood tests (data not shown). Histological assessment of colitis severity demonstrated a similar degree of inflammation both after 4 days of 3.5% DSS challenge (data not shown) as well as after 7 days of 3.5% DSS challenge (Fig. 1B). No histological signs of inflammation or weight loss were observed in water control treated animals (Fig. 1A and data not shown). Thus, CD137^−/−^ and WT mice develop a similar disease severity in acute 3.5% DSS-induced colitis.

### CD137^−/−^ Mice Show Increased Weight Loss during the Resolution Phase After a 2.0% DSS Challenge

We next investigated whether CD137 influences the resolution of intestinal inflammation. CD137^−/−^ and WT mice were given 2.0% DSS in drinking water ad libitum for a period of 5 days, after which time mice were switched back to water to allow subsiding of the colonic inflammation. At different time points after DSS withdrawal mice were sacrificed, the intestines were removed and sections were embedded for histological scoring of inflammation severity.

**Figure 2:**
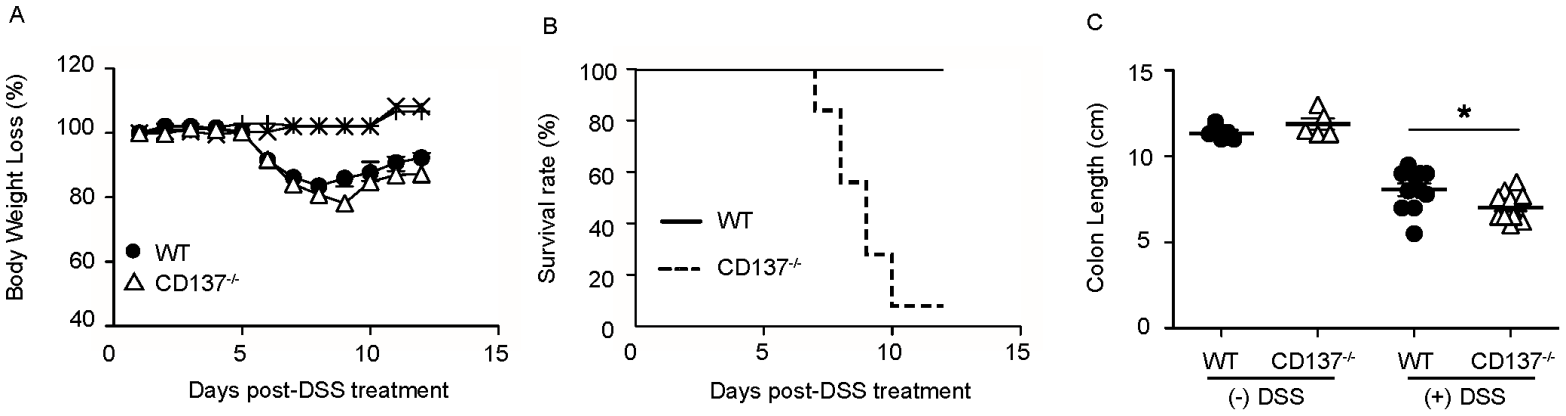
Increased susceptibility of CD137^−/−^ mice during the resolution phase after 5 days of 2.0% DSS treatment as compared to WT mice. A, Percentage of body weight loss over the course of 5 days of treatment with 2.0% DSS water followed by 7 days of normal water. B, Kaplan-Meier-Analysis using a combined end-point of weight-loss above 20% of original weight/death in 5 days 2.0% DSS treated mice followed by 7 days of recovery phase with normal water. C, Colon length of mice untreated or treated for 5 days with 2.0% DSS followed by 3 days of normal water. Data are representative of at least three independent experiments, shown here are means ± SEM, n = 10–15 for DSS groups, n = 5 for water control groups (* p<0.05).

CD137^−/−^ mice demonstrated an increased weight loss during the resolution phase of intestinal inflammation after DSS withdrawal compared to WT mice (Fig. 2A). Kaplan-Meier-Analysis using a combined end-point of weight-loss above 20% of original weight/death demonstrated a significantly increased drop-out rate in CD137^−/−^ mice as compared to WT mice after DSS withdrawal (Fig. 2B). Also the surviving CD137^−/−^ mice demonstrated significantly increased histological inflammation 7 days after DSS withdrawal compared to WT mice (data not shown). Besides histological examination, shortening of the colon can be used as a measure of inflammation. We found significant colonic shortening 3 days after DSS withdrawal in both CD137^−/−^ and WT mice as compared to untreated mice, and significantly shorter colons in CD137^−/−^ DSS-treated mice than in WT DSS-treated mice (Fig. 2C). These results indicate that resolution of intestinal inflammation is impaired in CD137^−/−^ mice.

### Resolution of Inflammation After DSS Withdrawal is Impaired in CD137^−/−^ Mice

To assess the development of inflammation after 2.0% DSS exposure, CD137^−/−^ and WT mice were divided into two groups each. One group of mice was sacrificed after 5 days of 2.0% DSS exposure, while the other group of mice was allowed to resolve intestinal inflammation for an additional 3 days on drinking water.

**Figure 3:**
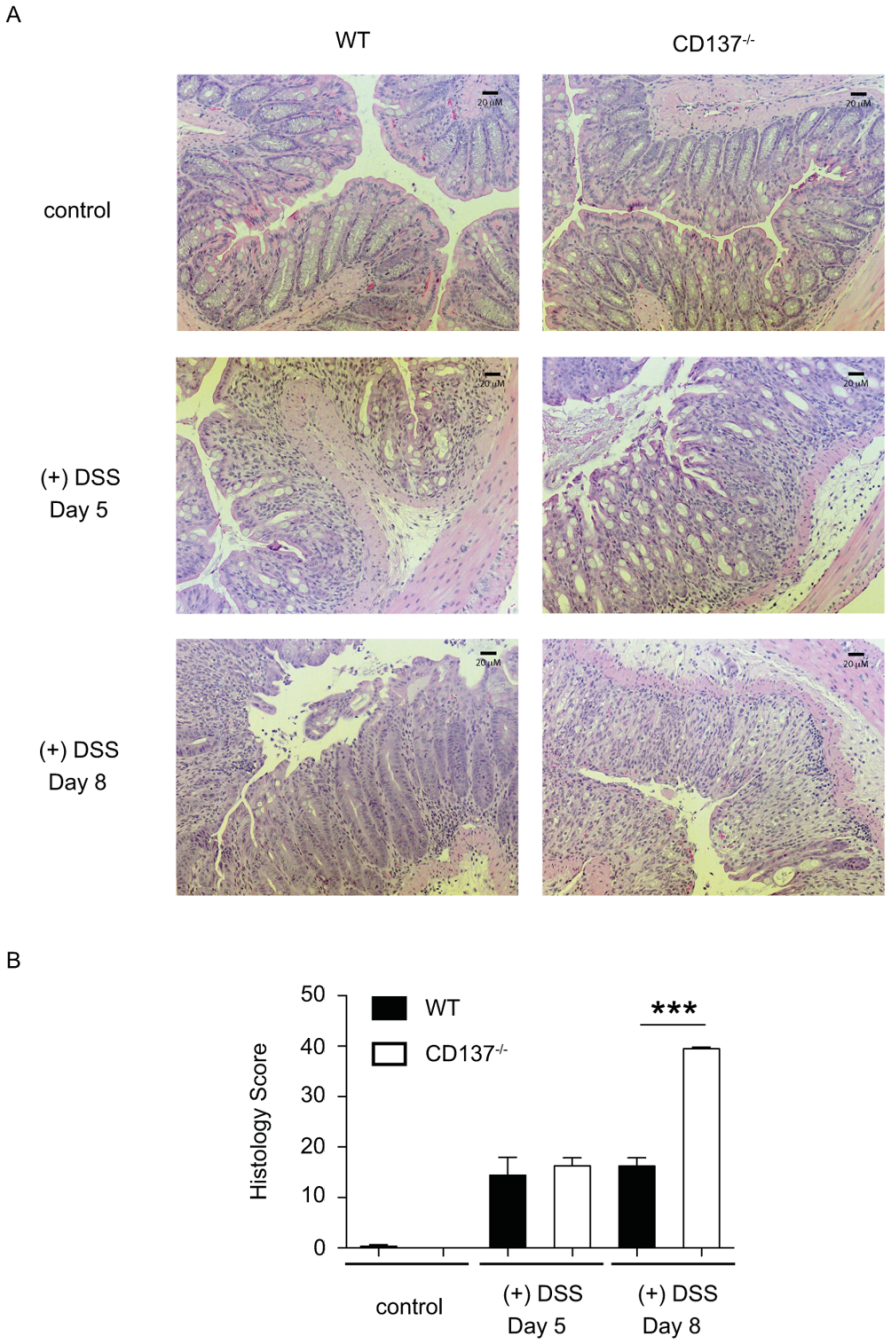
CD137^−/−^ and WT mice develop a similar histologic disease severity after 5 days of acute 2.0% DSS exposure, however, resolution of inflammation after withdrawal of DSS is impaired in CD137^−/−^ mice. A, Representative histologic colonic cross-sections of mice exposed to normal water (control), 5 days of 2.0% DSS water ((+)DSS Day 5) and 5 days of 2.0% DSS water followed by 3 days normal water ((+)DSS Day 8) stained with Hematoxylin-Eosin (20-fold magnification). B, Histology score of WT (black bars) and CD137^−/−^ (white bars) mice untreated, treated with 2.0% DSS for 5 days or mice treated with 2.0% DSS for 5 days followed by 3 days of normal water. Data are representative of two independent experiments, shown here are means ± SEM, n = 4 per group (*** p<0.001).

CD137^−/−^ and WT mice developed a similar degree of colitis severity after 5 days of 2.0% DSS exposure as assessed by histological criteria (Fig. 3A, B). However, while in WT mice inflammation did not progress after withdrawal of DSS, CD137^−/−^ mice showed a histological progression towards severe colonic inflammation (Fig. 3A, B). These histological results correlate with the increased weight loss and increased drop-out rate observed in CD137^−/−^ mice after 2.0% DSS exposure (Fig. 2A, B). In summary, CD137^−/−^ and WT mice develop a similar histologic disease severity after 5 days of acute 2.0% DSS exposure, however, resolution of inflammation after withdrawal of DSS is impaired in CD137^−/−^ mice.

### 2.0% DSS-induced Colitis Leads to Increased Local Colonic IFN-γ, GM-CSF, CXCL1 and IL-17 Secretion in CD137^−/−^ Mice

**Figure 4:**
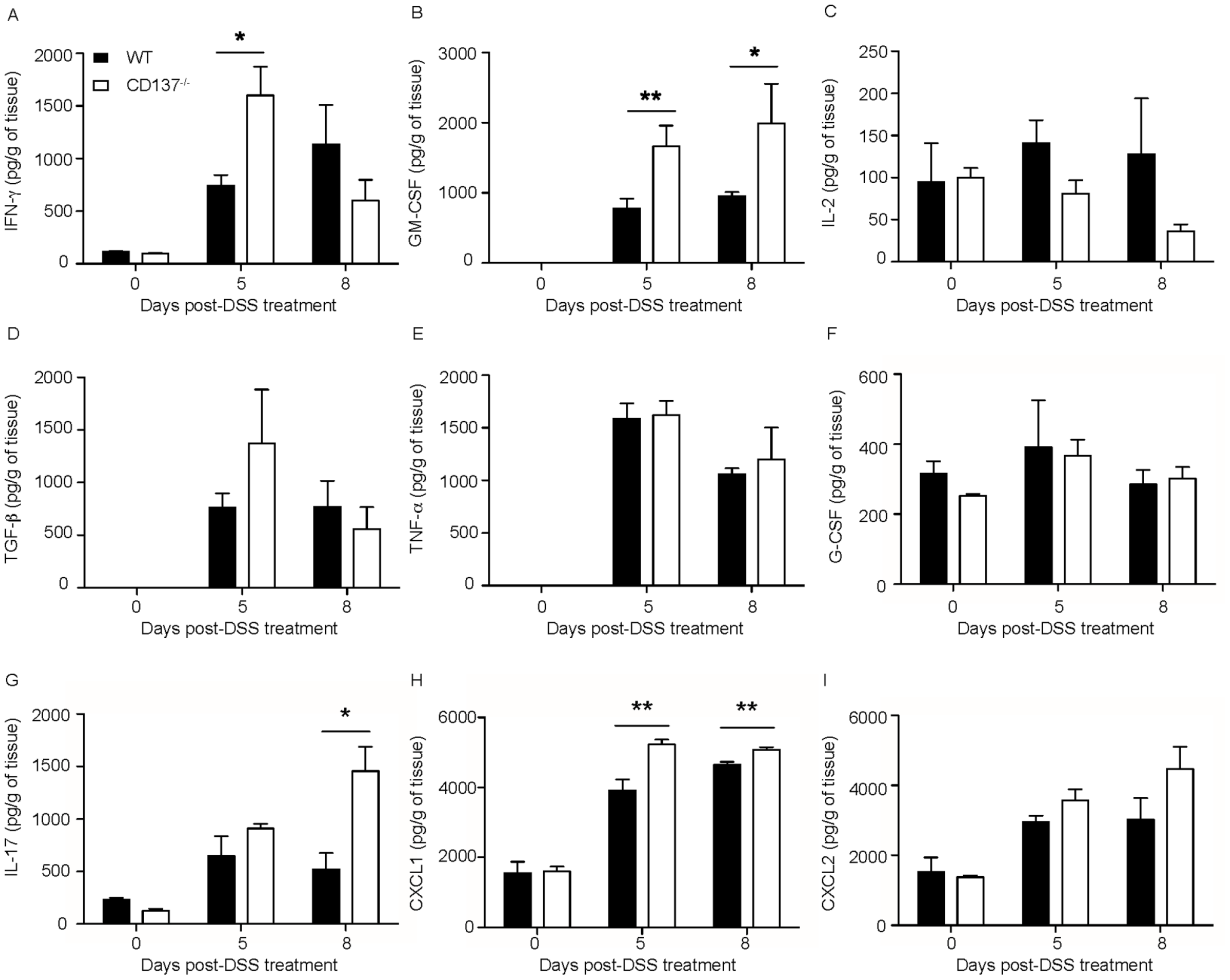
2.0% DSS-induced colitis leads to increased local colonic IFN-γ, GM-CSF, CXCL1 and IL-17 secretion in CD137^−/−^ versus WT mice. Colon segment culture supernatants of CD137^−/−^ (white bars) and WT mice (black bars) untreated (day 0), or treated either for 5 days with 2% DSS (day 5) or 5 days with 2% DSS followed by 3 days with normal water (day 8), were examined for IFN-γ (A), GM-CSF (B), IL-2 (C), TGF-§ (D), TNF-α (E), G-CSF (F), IL-17 (G), CXCL1 (H) and CXCL2 (I). Data are representative of two independent experiments, shown here are means ± SEM, n = 4 per group (* p<0.05, ** p<0.01).

Having observed a difference in the resolution of inflammation we investigated whether this is reflected and possibly attributable to a difference in the cytokine profile between CD137^−/−^ and WT mice. Thus, mice were exposed to 2.0% DSS for a period of 5 days, or to 5 days DSS plus an additional 3 days with normal water, after which time animals were sacrificed. Local cytokine secretion in colon segment cultures was then evaluated using previously published techniques [29], [30]. Interestingly, colon segments of CD137^−/−^ mice that were exposed for 5 days to 2.0% DSS exhibited increased IFN-γ secretion compared to colon segments of WT mice with the same treatment, indicating a stronger Th1-type colonic inflammatory response in the absence of CD137 (Fig. 4A). Additionally, the colon segments from the CD137^−/−^ mice had an increased GM-CSF, IL-17 and CXCL1 secretion which points to an increased pro-inflammatory neutrophil response (Fig. 4B, G, H). Of note, increased levels of IL-17 secretion after colitis induction indicate increased numbers of Th17 cells (Fig. 4G). Secretion of IL-2, which is important for growth, proliferation and differentiation of T cells, was reduced in colon segment cultures of 2.0% DSS-exposed CD137^−/−^ compared to WT mice, however, the difference did not reach statistical significance (Fig. 4C). The levels of TGF-ß, TNF-α, G-CSF and CXCL2 did not significantly vary between the two mouse strains (Fig. 4D–F, I). It should be emphasized that the cytokine secretion profiles were observed after 5 days of 2.0% DSS-exposure, at a time when histologically there were no differences detectable in the severity of the colonic inflammatory response (Fig. 3A, B).

Of note, no differences were observed in systemic cytokine profiles (IL-6, IL-10, IL-13, IL-17A, IFN-γ, TNF-α, GM-CSF) as measured in whole blood drawn after 5 days of 2.0% DSS-exposure as well as 8 days and 11 days post DSS treatment (data not shown). In summary, 2.0% DSS-induced colitis is accompanied by increased local colonic IFN-γ, GM-CSF, CXCL1 and IL-17 secretion in CD137^−/−^ compared to WT mice.

### CD137^−/−^ Mice have Increased Numbers of Neutrophils during the Resolution of DSS-induced Inflammation

In addition to the altered cytokine profile we asked whether the different histopathology observed in WT and in CD137^−/−^ mice was accompanied by a change in the infiltrating leukocyte subsets in the colonic mucosal lamina propria. The specific subsets of lamina propria mononuclear (inflammatory) cells contributing to the increased DSS-induced colitis were characterized in mice that received 5 days of 2.0% DSS treatment, followed by a resolution phase of 3 days. On days 5 and 8, mice were sacrificed, the large intestines were removed and the intestinal lymphocytes from both epithelial (IEL) and lamina propria (LPMNC) compartments of the large intestine were isolated as described previously [31], [32], and were analyzed by flow cytometry.

**Figure 5:**
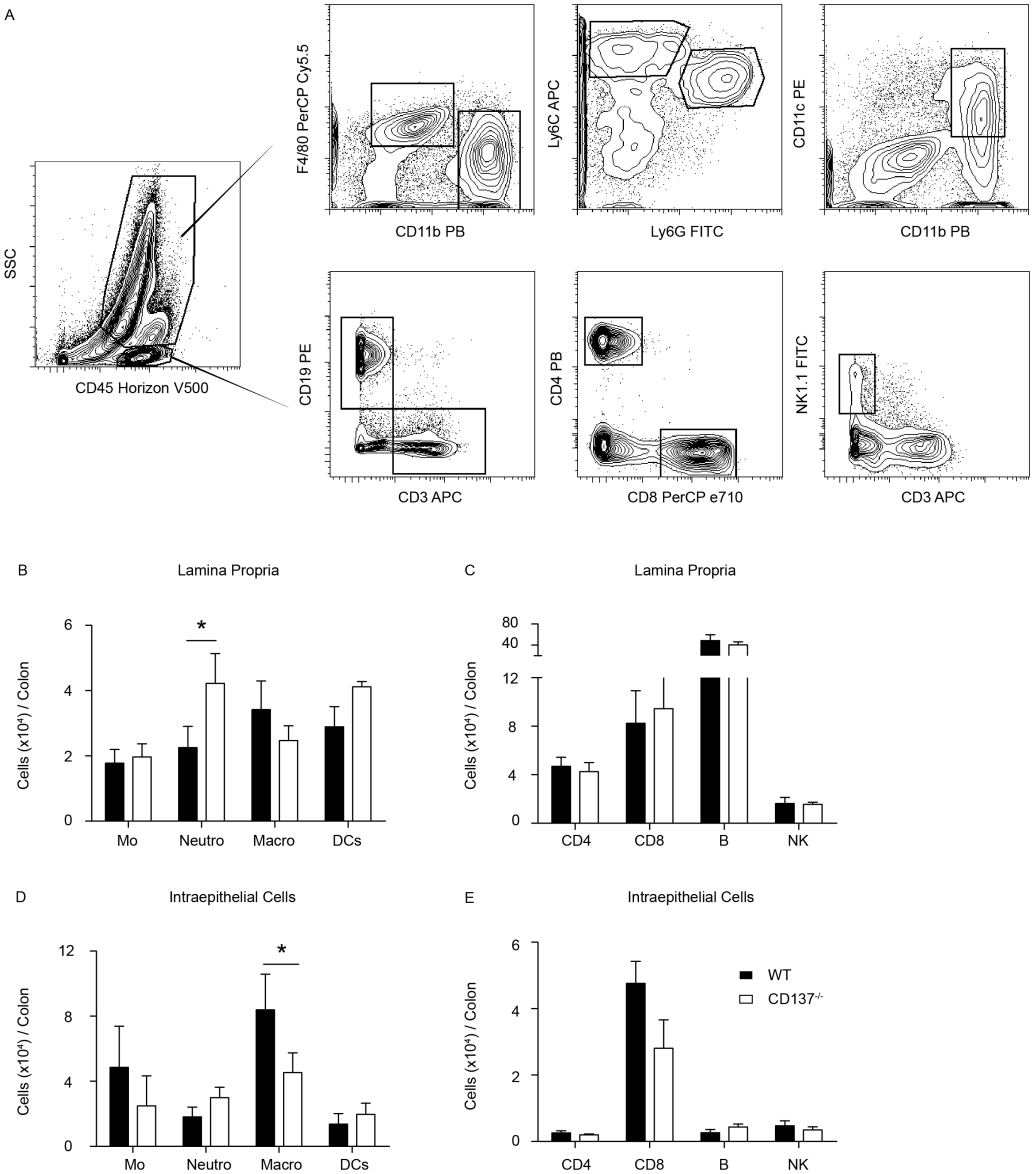
CD137^−/−^ mice have increased numbers of neutrophils in the lamina propria mononuclear cell (LPMNC) subpopulations while presenting lower macrophage numbers in the intraepithelial (IEL) subpopulation. A, Gating strategy for myeloid (top panel) and lymphocyte (lower panel) populations. B, Quantification of different immune cell populations in the LPMNC cells (B, C) and IEL cells (D, E) during the resolution phase of inflammation 3 days after withdrawal of 2.0% DSS. Data represent means ± SEM of 4 independent experiments pooled together, n = 3–10 per group (* p<0.05). Mo (Monocytes), Neutro (Neutrophils), Macro (Macrophages), DCs (Dendritic cells), NK (Natural Killer cells).

There was minimal cellular infiltrate with no difference in the subsets of intestinal lamina propria mononuclear cells (LPMNC) in CD137^−/−^ versus WT mice 5 days after 2.0% DSS exposure (data not shown). However, 3 days after withdrawal of DSS, CD137^−/−^ mice had significantly increased numbers of neutrophilic granulocytes in the LPMNC subset, reflecting the increased inflammation (Fig. 5A, B). At the same time, CD137^−/−^ mice had significantly decreased numbers of macrophages in the IEL subset compared to WT mice (Fig. 5A, D). Since macrophages have been demonstrated to be necessary for intestinal regenerative responses to injury [33], these observations suggest an impaired colonic restitution in CD137^−/−^ mice. No differences were observed in the numbers of monocytes, B cells, NK cells, dendritic cells, CD4^+^ or CD8^+^ T cells, neither among the LPMNC (B, C) or the IEL (D, E), nor in untreated CD137^−/−^ versus WT mice (data not shown). In summary, the decreased number of macrophages that are necessary for mucosal healing, together with the increased recruitment of neutrophils to the lamina propria of CD137^−/−^ mice may explain the increased severity of DSS-colitis in these mice.

## Discussion

The current study investigated DSS-induced colitis in CD137^−/−^ and WT mice. We found that in the absence of CD137, mice develop a more severe colitis, suggesting that CD137 exerts an anti-inflammatory effect in this model.

Of note, both CD137^−/−^ and WT mice demonstrated a similar degree of inflammation after 5 days of DSS exposure. However, the resolution of colonic inflammation was significantly impaired in CD137^−/−^ mice. This led to prolonged tissue infiltration of leukocytes, an increased neutrophil/macrophage ratio among the infiltrating cells and an increased release of the pro-inflammatory cytokines IFN-γ, GM-CSF, CXCL1 and IL-17.

The reason for the more pronounced disease manifestation in the CD137^−/−^ mice may be due to several factors. Overexpression of the granulocyte survival factor GM-CSF has been associated with suppression of neutrophil apoptosis in the absence of CD137 activation [34]. It has been shown in vitro that CD137 is constitutively expressed on neutrophilic granulocytes, and that the CD137 signaling pathway blocks the anti-apoptotic effect mediated by the §-subunit of the GM-CSF receptor in neutrophils [35]. Therefore, the more severe disease manifestation in the CD137^−/−^ mice may be due to the absence of CD137-mediated granulocyte apoptosis leading to increased granulocyte accumulation and subsequent tissue damage, since mucosal inflammation in DSS-induced colitis is mediated by neutrophilic granulocytes [36], [37]. In line with this hypothesis and published data, we found increased GM-CSF in the colons of CD137^−/−^ mice, which likely supported neutrophil accumulation in the colons. Additionally, we found increased levels of CXCL1 in colitic CD137^−/−^ mice, which increase neutrophil influx into the inflamed mucosal tissue. Remarkably, increased levels of IL-17 in CD137^−/−^ mice indicate increased numbers of Th17 cells, which is further supporting neutrophil recruitment [38]. Although the initial infiltration by neutrophils is beneficial for killing bacteria, it is presumed that it is the persistent presence of neutrophils in the tissues that causes damage [36], [37]. Of note, it has recently been demonstrated in a T-cell transfer model of colitis that GM-CSF skews hematopoietic stem and progenitor cells towards granulocyte-monocyte progenitor cells, leading to the exacerbation of intestinal inflammation. Importantly, colitis could be ameliorated by GM-CSF blockade [39]. Thus, the GM-CSF driven increased accumulation and prolonged presence of neutrophils in the colons of CD137^−/−^ mice can explain the increased tissue damage in these mice.

There is an additional function of CD137, whose absence in the CD137^−/−^ mice is likely to contribute to the more severe disease manifestation: CD137 not only regulates the activities of mature leukocytes, it also regulates hematopoiesis, and has been shown to induce proliferation and expansion of hematopoietic progenitor cells. Concomitantly, CD137 induces myeloid differentiation, and within the myeloid lineage monocytic differentiation [40], [41], especially during inflammation [42]. In fact, CD137 and G-CSF compete for driving the differentiation of myeloid progenitor cells into the monocytic and granulocytic differentiation, respectively, with an increasing G-CSF/CD137 ratio leading to a preponderance of granulocytes [43]. In the absence of CD137 more myeloid precursor cells would be expected to differentiate to granulocytes at the expense of monocytes and macrophages. Indeed, we not only see an increase of granulocytes in the colons CD137^−/−^ compared to WT mice as a response to DSS administration but also a decrease in macrophages. This increased granulocyte/macrophage ratio could not only contribute to the more severe colitis in the CD137^−/−^ mice through recruiting more granulocytes that in turn cause tissue damage but also because macrophages are the cells that coordinate wound healing [33]. With fewer macrophages being present in the colons of the CD137^−/−^ mice, resolution of inflammation and wound healing are expected to be delayed, and that is what we observe.

The CD137 receptor/ligand system has so far been mainly associated with proinflammatory effects. Agonistic anti-CD137 antibodies costimulate T cells, and enhance immunity leading to rejection of tumors and transplants in mice, and enhance anti-virus immune responses [5], [44], [45], [46]. Further, bidirectional signaling exists for the CD137 receptor/ligand system, and reverse signaling of CD137L activates antigen presenting cells, which leads to a further enhancement of immune responses [47]. The proinflammatory activities of CD137 and CD137L are also evident in autoimmune diseases. The administration of antagonistic anti-CD137 ligand antibodies to mice with collagen-induced arthritis resulted in a milder disease [48]. Further, experimental autoimmune encephalomyelitis is more severe in mice that are deficient in CD137L [49]. However, the very same agonistic anti-CD137 antibodies that enhance anti-tumor immunity have been shown to quench autoimmunity and to ameliorate autoimmune diseases, including experimental autoimmune encephalomyelitis and type 1 diabetes [8], [9], [10], [50], [51]. An explanation for these puzzling findings and seemingly contradictory activities has not yet been found. These examples demonstrate that the consequences of modulating the CD137 receptor/ligand system are difficult to predict in autoimmune diseases. Nevertheless, our findings in the DSS colitis model suggest that neutralizing CD137 with antagonistic anti-CD137 antibodies would not be beneficial for the treatment DSS-induced colitis and possibly also not for IBD patients. Our data suggest that agonistic anti-CD137 antibodies may be more suited to ameliorate colitis.
