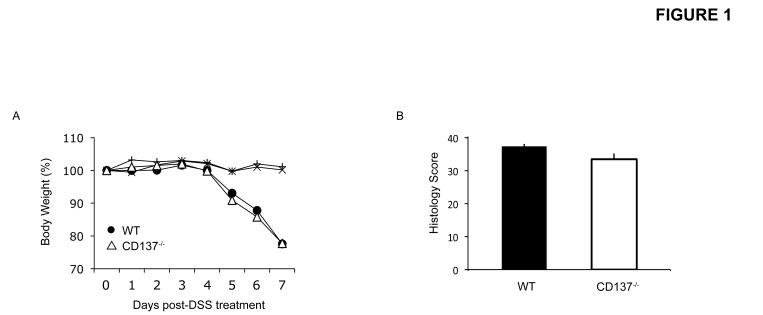# Correction: CD137 Facilitates the Resolution of Acute DSS-Induced Colonic Inflammation in Mice

**DOI:** 10.1371/annotation/83fd162d-b115-4af3-b6e7-dc23ae3f9a76

**Published:** 2014-01-22

**Authors:** Julia M. Martínez Gómez, Lieping Chen, Herbert Schwarz, Thomas Karrasch

An error to the y-axis of Figure 1B was introduced during production of this article. Please see the corrected Figure 1 here: 

**Figure:**